# Partial nephrogenic diabetes insipidus associated with Castleman’s disease

**DOI:** 10.1186/s12882-019-1343-9

**Published:** 2019-05-14

**Authors:** Minah Kim, Hong Sang Choi, Eun Hui Bae, Seong Kwon Ma, Soo Wan Kim, Chang Seong Kim

**Affiliations:** Department of Internal Medicine, Chonnam National University Medical School, 42 Jebongro, Gwangju, 61469 Republic of Korea

**Keywords:** Castleman’s disease, Obstructive uropathy, Nephrogenic diabetes insipidus

## Abstract

**Background:**

Nephrogenic diabetes insipidus (DI) secondary to a urinary tract obstruction is a rare condition. Herein, we report a case of partial nephrogenic DI due to obstructive uropathy in a patient with Castleman’s disease.

**Case presentation:**

A 78-year-old man underwent computed tomography (CT) at his local hospital because of persistent edema of the leg and polyuria (both lasting approximately 2 months); retroperitoneal fibrosis was detected on the CT scan. An abdominal CT scan showed bilateral hydronephrosis, and a surgical biopsy of the para-aortic lymph node revealed Castleman’s disease. To resolve the hydronephrosis, a double J stent was inserted; however, his polyuria continued. As his serum osmolality (311 mOsm/kg) was greater than 300 mOsm/kg, and his serum sodium level was 149 mEq/L, a water deprivation test was not performed. On a vasopressin challenge test, his urine was not sufficiently concentrated to the expected range, indicating partial nephrogenic DI. He was treated with hydrochlorothiazide (25 mg/day), and his urine output gradually decreased to within the normal range. The patient recovered uneventfully and underwent treatment for Castleman’s disease.

**Conclusion:**

To the best of our knowledge, this is the first case of partial nephrogenic DI due to obstructive uropathy associated with Castleman’s disease.

**Electronic supplementary material:**

The online version of this article (10.1186/s12882-019-1343-9) contains supplementary material, which is available to authorized users.

## Background

Diabetes insipidus (DI) is a syndrome characterized by the chronic excretion of abnormally large volumes of dilute urine. There are four types of DI: central (pituitary) DI, primary polydipsia, gestational DI, and nephrogenic DI. In central DI, the antidiuretic hormone (ADH) level is lower than normal. Diseases of the hypothalamus-pituitary gland axis may cause central DI. Primary polydipsia is due to the suppression of arginine vasopressin (AVP) secretion by excessive fluid intake, and gestational DI is caused by a deficiency of plasma AVP, resulting from increased hormone degradation by an enzyme made in the placenta [1]. In contrast, nephrogenic DI results from a failure of the kidneys to concentrate the urine due to an insensitivity of the distal nephron to the antidiuretic hormone, AVP.

Nephrogenic DI can be classified as congenital or acquired [2]. Acquired nephrogenic DI has been described in a number of clinical settings; however, nephrogenic DI secondary to a urinary tract obstruction is a rare clinical problem. This condition was first reported in 1954 by Roussak and Oleesky [3]. Ureteral obstruction may result from stones, a transitional cell carcinoma, or external compression (by tumors, enlarged lymph nodes, or retroperitoneal fibrosis).

In 1956, Castleman et al. described an entity involving localized mediastinal lymph node hyperplasia, resembling a thymoma [4]. Castleman’s disease, which is diagnosed as retroperitoneal fibrosis with hydronephrosis, has rarely been reported. Herein, we report the first known case of partial nephrogenic DI with hydronephrosis in a patient with Castleman’s disease.

## Case presentation

**Fig. 1:**
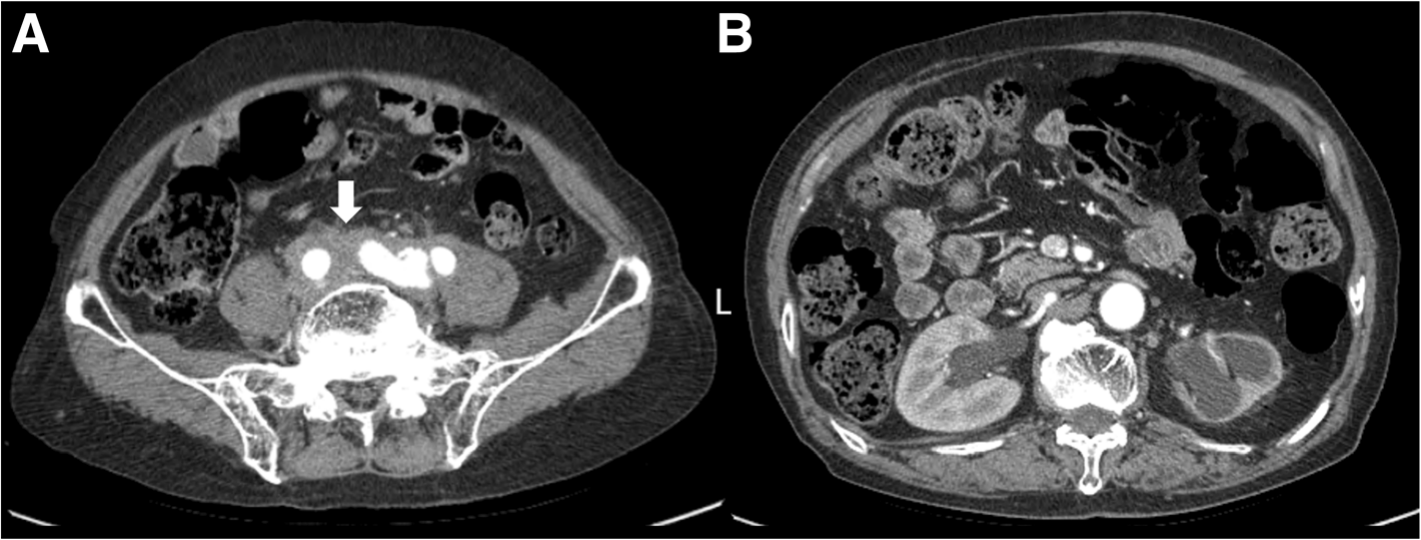
Abdominal computed tomography (CT). **a** The retroperitoneal mass surrounding the vessles ranging from the aortic bifurcation to presacral area (arrow). **b** Both hydronephrosis and atrophied left kidney

A 78-year-old man was admitted to a local hospital because of leg swelling and polyuria (both lasting for approximately 2 months). He had not received any medication for his complaint. He had no known medical history of hypertension, diabetes mellitus. Physical examination showed the patient to be well nourished but he craved for water. Retroperitoneal fibrosis was detected on computed tomography (CT). The retroperitoneal fibrotic tissue was compressing both distal ureters, and bilateral hydronephrosis was identified on CT (Fig. 1a and b). To resolve the hydronephrosis, a double J stent was inserted in the right-side ureter; however, insertion into the left-side ureter failed due to atrophy. To evaluate the cause of the retroperitoneal fibrosis, we decided to perform a laparoscopic surgical biopsy. Because the retroperitoneal fibrotic tissues were too stiff for biopsy, we biopsied the para-aortic lymph node, just adjacent to the fibrotic tissue. The biopsy test results showed the classical characteristics of Castleman’s disease (hyaline-vascular type, negative for human herpesvirus 8).

Before the initiation of radiation treatment, he still complained of frequent urination (20 times/day), excessive thirst (visual analogue scale 8) [5], and his urine output was approximately 5~6 L/day. His baseline biochemical levels were as follows: blood urea nitrogen, 19.6 mg/dL; creatinine, 1.4 mg/dL; sodium, 149 mEq/L; potassium, 4.8 mEq/L; chloride, 118 mEq/L; serum osmole, 311 mOsm/kg; and random glucose, 131 mg/dL. On urine analysis, the specific gravity was under 1.005, representing diluted urine, and the urine osmolality was 148 mOsm/kg. No protein, glucose, or red blood cells were seen on a urine analysis. A basal plasma AVP level was 5.24 pg/ml, which was above the normal range (0~4.7 pg/ml). An antinuclear antibody test was performed to further evaluate the retroperitoneal fibrosis. The antinuclear antibody test was positive, with a homogeneous pattern, but the specific tests for extractable nuclear antigen antibodies and double-strand deoxyribonucleic acid were negative.

**Fig. 2:**
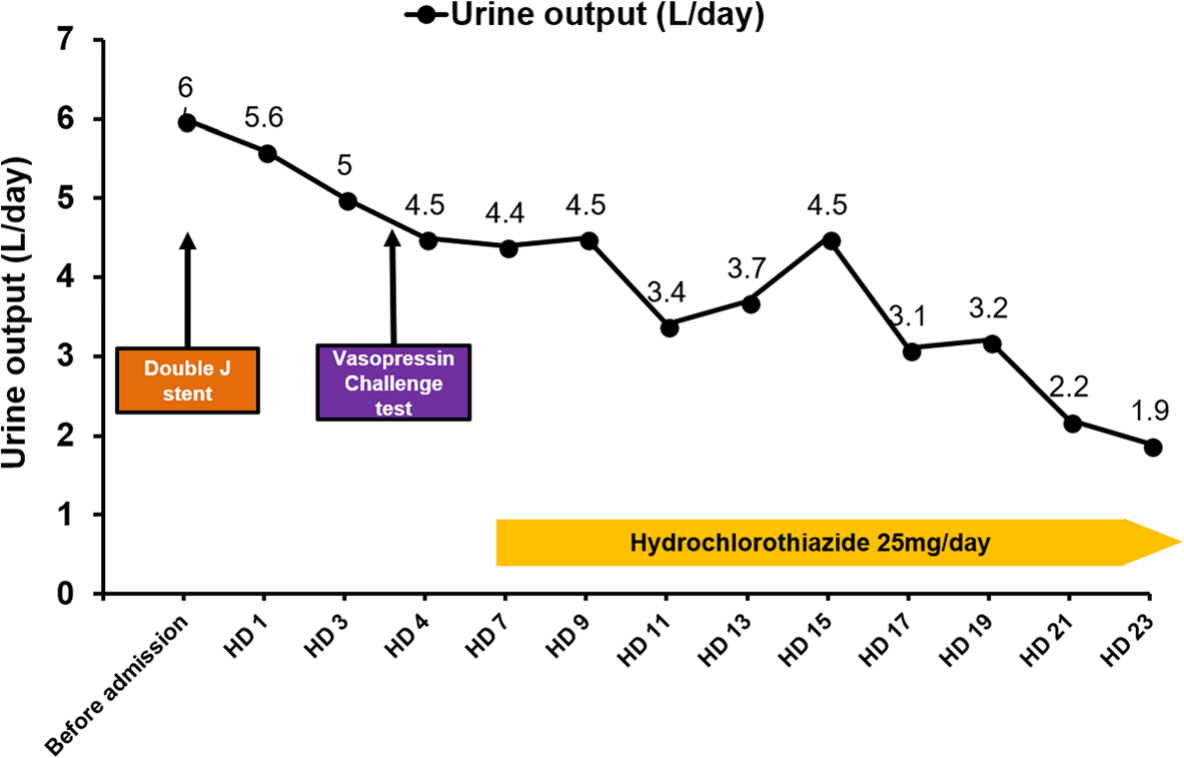
Change in urine volume during hospitalization. HD, Hospital day

A water deprivation test was not performed, as his serum osmolality (311 mOsm/kg), and serum sodium (149 mEq/L) were above the threshold for maximal AVP secretion (serum osmolality, 300 mOsm/kg; serum sodium, 145 mEq/L). Thus, we performed a vasopressin challenge test (Additional file 1: Figure S1). After the vasopressin injection, his urine osmolality increased to 206 mOsm/kg, which was approximately 39% greater than that at baseline (before the vasopressin injection: 148 mOsm/kg). Thus, the urine was not sufficiently concentrated to the expected range, indicating partial nephrogenic DI. Partial nephrogenic DI can be diagnosed as a small elevation (up to 45%) in urine osmolality after a vasopressin injection, with the urine osmolality remaining well below isosmotic urine. Compared to that in patients with partial nephrogenic DI, patients with partial central DI usually achieve a urine osmolality of 300 mOsm/kg or higher after a vasopressin injection [6, 7]. After the vasopressin challenge test, he still complained with remained thirst (visual analogue scale 4). We treated the patient with hydrochlorothiazide (25 mg/day), and his urine output gradually decreased to within the normal range (Fig. 2). After treatment of hydrochlorothiazide, his serum sodium level recovered to between 138 and 142 mEq/L. The patient recovered uneventfully and underwent treatment for Castleman’s disease in our hematology department.

## Discussion

Nephrogenic DI is defined as a reduction in urinary concentrating ability, caused by a resistance to the actions of ADH. Patients with an early or mild form of nephrogenic DI show little or no symptoms, as they compensate for their fluid loss with increased water intake. Patients with moderate to severe nephrogenic DI present with polyuria, nocturia, and polydipsia. In severe cases, uncompensated symptoms can develop into marked dehydration, neurologic symptoms, and encephalopathy [2].

Nephrogenic DI can be congenital or may be acquired, as in the present case. Hereditary nephrogenic DI is a common cause of nephrogenic DI in children, while chronic lithium ingestion and hypercalcemia are common causes of nephrogenic DI in adults. Acquired causes are often at least partially reversible, with cessation of the offending drug or correction of the hypercalcemia [8]. Chronic disorders, such as sickle cell disease and kidney failure, can damage the kidney’s ability to respond to ADH [9]. Chronic partial urinary tract obstruction is a rare cause of nephrogenic DI. In two previous reports of such cases, the obstructive uropathy was due to a malignancy (advanced stage ovarian cancer and prostate cancer) [10, 11]. In these previous cases, the patients’ urine output and urine osmolality recovered to normal ranges after surgery and thiazide treatment [10, 11]. Similarly, the urine output and urine osmolality recovered to normal ranges after treatment in the present case.

The exact cause of the nephrogenic DI in the above-mentioned cases of chronic obstructive uropathy remains unclear, but it is likely that increased pressures on the collecting ducts caused damage to the tubular epithelium, resulting in a resistance to the actions of ADH [10]. In addition, the direct suppression of aquaporin-2 has been observed in animal models of urinary tract obstruction, which might also explain the mechanism [12].

Castleman’s disease comprises a heterogeneous group of lymphoproliferative disorders, with unicentric and multicentric forms. Several cases of Castleman’s disease have been reported in patients with retroperitoneal fibrosis. For example, Massaru et al. [13] reported eight cases of Castleman’s disease of the retroperitoneum. In 3 of the cases, the retroperitoneum was involved, and in 5 of the cases, the ureter and renal hilum were involved [13]. In addition, there are several case reports of hydronephrosis associated with Castleman’s disease involving the ureter [14–16]. However, no cases of partial nephrogenic DI with hydronephrosis caused by Castleman’s disease have been previously reported. Thus, the present case is very unique.

## Conclusion

In conclusion, our patient had unicentric Castleman’s disease of the hyaline-vascular type, with an initial presentation of bilateral hydronephrosis (identified on CT). Our patient had unique features, presenting with polyuria and polydipsia, and was diagnosed with partial nephrogenic DI. This is the first case of partial nephrogenic DI with hydronephrosis caused by Castleman’s disease. Given the rarity of the nephrogenic DI associated with hydronephrosis and Castleman’s disease, it is difficult to speculate on the possible associations in a clinical setting. However, if polyuria is present in a patient with Castleman’s disease accompanied by retroperitoneal fibrosis, evaluations of obstructive uropathy and nephrogenic DI should be performed.

## Additional file

Additional file 1: **Figure S1.**Vasopressin challenge test. (TIF 4946 kb)

## Abbreviations

ADH: Antidiuretic hormone
AVP: Arginine vasopressin
CT: Computed tomography
DI: Diabetes insipidus
