# Supplementary figures and images for: Partial nephrogenic diabetes insipidus associated with Castleman’s disease
Source: BMC Nephrol. 2019 May 14;20:168. doi: 10.1186/s12882-019-1343-9 (PMC6518727; doi:10.1186/s12882-019-1343-9)

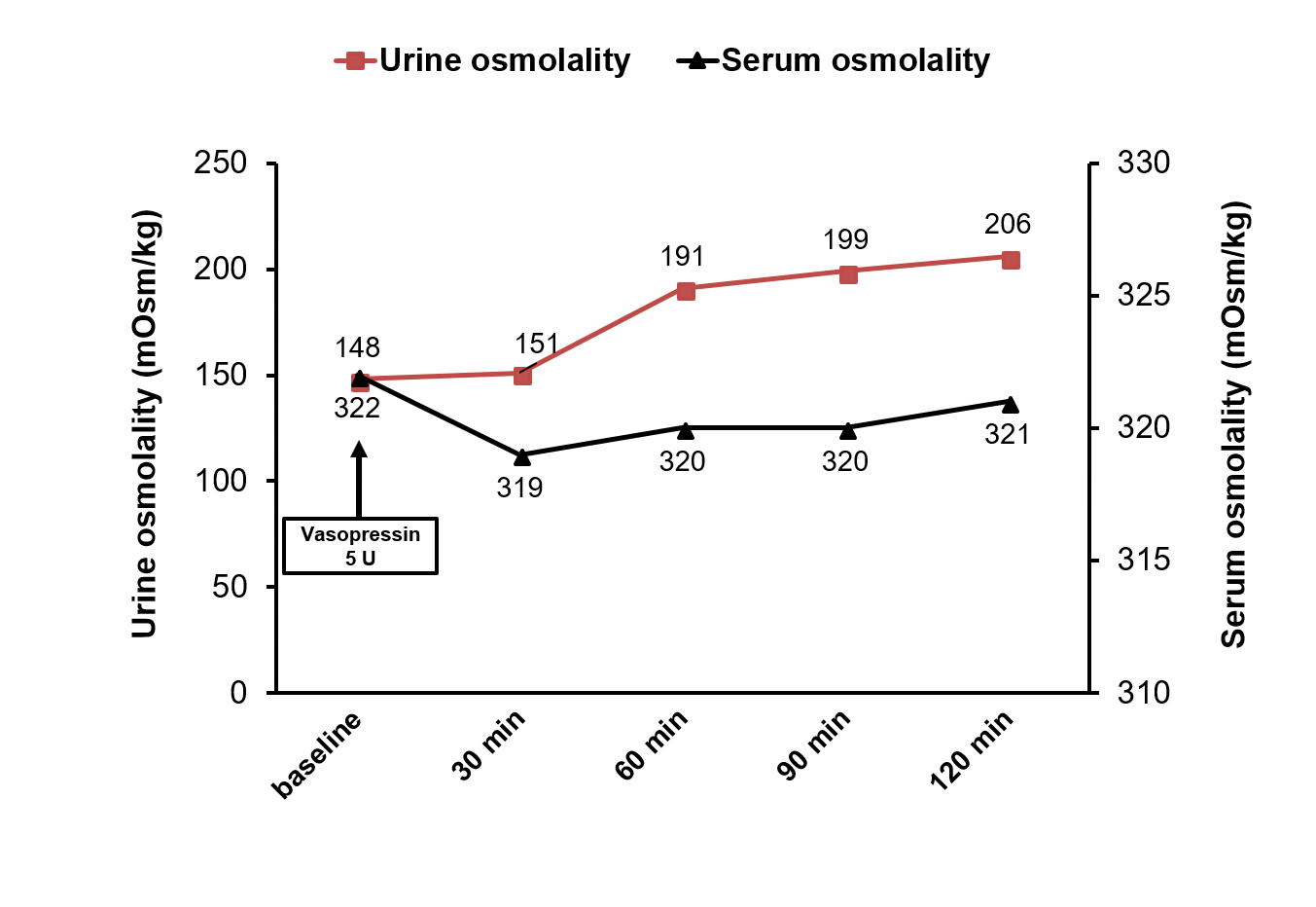

Supplement: Supplementary file 1 — Figure S1.Vasopressin challenge test. (TIF 4946 kb) [file 12882_2019_1343_MOESM1_ESM.tif]
